# Goose Mx and OASL Play Vital Roles in the Antiviral Effects of Type I, II, and III Interferon against Newly Emerging Avian Flavivirus

**DOI:** 10.3389/fimmu.2017.01006

**Published:** 2017-08-23

**Authors:** Shun Chen, Wei Zhang, Zhen Wu, Jinyue Zhang, Mingshu Wang, Renyong Jia, Dekang Zhu, Mafeng Liu, Kunfeng Sun, Qiao Yang, Ying Wu, Xiaoyue Chen, Anchun Cheng

**Affiliations:** ^1^Institute of Preventive Veterinary Medicine, Sichuan Agricultural University, Chengdu, China; ^2^Research Center of Avian Disease, College of Veterinary Medicine, Sichuan Agricultural University, Chengdu, China; ^3^Key Laboratory of Animal Disease and Human Health of Sichuan Province, Sichuan Agricultural University, Chengdu, China

**Keywords:** goose interferons, Tembusu virus, antiviral response, RNA sequencing, Mx, OASL

## Abstract

Duck Tembusu virus (TMUV), an emerging avian flavivirus, is highly pathogenic to birds and has the potential to become a zoonotic pathogen. Here, the molecular antiviral mechanism of goose type I, II, and III interferon (goIFNα, goIFNγ, and goIFNλ), the key components of the innate immune pathway, against TMUV was studied. We found that the transcription of goIFNs was obviously driven by TMUV infection *in vivo* and *in vitro*, and the titers and copies of TMUV were significantly reduced following treatment with goIFNs. The results of RNA sequencing (RNA-seq) revealed that goIFN stimulation triggered a set of differentially expressed genes at different levels and a positive regulatory feedback loop of IFN release against infection. Two important interferon-stimulated genes, goMx and goOASL, were identified as workhorse IFNs in the inhibition of TMUV replication. The antiviral effects of goMx and goOASL were confirmed by transient overexpression and knockdown assay *in vitro*. Overall, our findings defined that goose Mx and OASL play key roles in the antiviral effects of type I, II, and III interferon against the TMUV. These results extend our understanding of the transcriptional profile of the goose IFN-mediated signaling pathway and provide insight into the antiviral mechanism of goIFNs against flavivirus infection.

## Introduction

In the classical innate immune pathway, incoming viral pathogens are sensed by cytologic host pattern recognition receptors (such as toll-like receptors, RIG-I-like receptors, NOD-like receptors), which activates downstream interferon regulatory factors (IRFs), leading to subsequent IFN production (1, 2). Interferons (IFNs) are cytokines with important antiviral activities and represent a powerful barrier to viral infection (3, 4); IFNs bind their cognate receptors and subsequently initiate a signaling cascade through the Janus kinase signal transducer and activator of transcription (JAK-STAT) pathway, triggering the expression of hundreds of IFN-stimulated genes (ISGs) (5, 6). These ISGs are mainly classified into three groups, positive regulators, negative regulators, and antiviral effectors, based on the numerous regulation mechanisms of innate immune signaling (7). As components of the IFN pathway, positive regulators (e.g., IRF1, cGAS) can promote IFN signaling and the subsequent development of a positive regulatory feedback loop. A small subset of negative regulators, such as SOCS1 and USP18, have the ability to target PRR, IRFs, or JAK/STAT to dampen the response and may even promote or enhance the replication of certain viruses (3, 8–11). Some ISGs were discovered as antiviral effectors that lead to a remarkable antiviral state, effectively intervening in distinct stages of the virus life cycle (12). It is well known that Mx1, OAS1, and PKR are potent antiviral effectors involved in the inhibition of viral infection (13–15).

Duck Tembusu virus (TMUV) is an emerging avian flavivirus, leading to a decrease in heavy egg laying, a sudden decline in feed uptake, and neurological signs in infected egg laying and breeder ducks (16, 17). According to incomplete statistics, multiple types of ducks (Pekin ducks, Cherry Valley ducks, Shaoxing ducks, Jinyun ducks, Longyan ducks, Jinding ducks, Khaki-Campbell ducks, Muscovy ducks, and Domesticated Mallards) have been subjected to TMUV infection (18). In addition to avian cell lines (DEF, GEF, and DF-1), TMUV can infect a range of mammalian cell lines (Vero, BHK21, HeLa, HepG2, and SH-SY5Y) and mosquito cell lines (C6/36 and *Aedes albopictus*) (19–21). These findings highlight the potential risk of TMUV infection in immunocompromised individuals and warrant studies on cross-species transmission and the pathogenesis of this novel flavivirus (21). Accumulating evidence suggests that IFN-regulated genes or ISGs (RIG-I, MDA5, TLR3, Mx, OAS, PKR, IFN-α, IFN-β, and IFN-γ, etc.) are induced to vastly different levels during TMUV infection in CEF, GEF, and 293 T cells (22–25). Recent studies have verified that TMUV infection triggers the IFN response mainly through MDA5 and TLR3-dependent signaling pathways (26). The expression of duMAVS was significantly upregulated after infection with TMUV, and the overexpression of duMAVS could drive the transcription of IFN-β, NF-κB, and IRF 7, as well as many downstream factors (such as Mx, PKR, OAS, and IL-8), which may partly explain the various viral replications that were significantly reduced by treatment with duMAVS (27). Moreover, RNA-seq analysis of the TMUV-infected goose spleen indicated that goose IFNs were key mediators of the host defense response to TMUV infection (unpublished data). These findings suggested that the IFN signaling pathway was essential in eliciting innate immune responses that restrict TMUV infection, and highly induced genes might be considered good candidates for controlling TMUV replication. However, the molecular antiviral mechanism of IFN against TMUV infection is still unknown.

With those factors in mind, here, we first investigated the expression of goose interferons (IFNα, IFNγ, and IFNλ) after TMUV infection *in vivo* and *in vitro*, as well as the antiviral activity of goIFNs against TMUV *in vitro*. Then, deep RNA sequencing (RNA-seq) (NCBI GEO accession number: GSE101404) was carried out to generate a high-resolution transcriptome map for IFN-treated PBMCs, and subsequently, some ISGs that might be involved in the IFN-dependent antiviral response were identified. Additionally, RNA interference (RNAi) assays were employed to confirm that goMx and goOASL play key roles in the antiviral effects of goIFNs against TMUV. Collectively, these studies provided evidence that the replication of TMUV was controlled through goIFNs, which initiated a positive regulatory feedback loop associated with antiviral and innate immune cellular signaling pathways.

## Materials and Methods

### Ethics Statement

The animal studies were approved by the Institutional Animal Care and Use Committee of Sichuan Agricultural University (No. XF2014-18) and followed the National Institutes of Health guidelines for the performance of animal experiments.

### Cells and Virus

Blood from geese was collected aseptically with heparin sodium (25 IU/mL). Immediately, PBMCs were isolated with the Goose Lymphocyte Separation Medium Kit (GLSMK) (TBD Sciences, Tianjing, China) according to the manufacturer’s protocol. Subsequently, cells (1 × 10^8^ cells/mL) were maintained in Gibco^®^ RPMI 1640 (Gibco Life Technologies, Shanghai, China) supplemented with 10% fetal bovine serum (FBS). Goose embryo fibroblasts (GEFs) and BHK-21 cells were grown supplemented with 10% FBS and maintained in Dulbecco’s Modified Eagle’s Medium (DMEM) (Gibco Life Technologies, Shanghai, China). All cells were cultured in 6-well plates at 37°C, 5% CO_2_. The duck TMUV CQW1 strain (GenBank Accession: KM233707) was isolated by our laboratory (28), and the measured virus titer was 6.3 × 10^6^ TCID_50_/100 μL, which was reported previously (25).

### Animal Experiments and Immunohistochemistry Assay (IHC)

Three-day-old Chinese geese (*Anser cygnoides*) were purchased from the breeding center of Sichuan Agricultural University (Yaan, Sichuan Province). The goslings were infected with TMUV (i. m. 500 µL) served as the experimental group and those treated with 500 µL of 0.9% NaCl served as the control group. From 1 to 4 dpi, the immune-associated tissues, such as the spleen (SP), liver (LI), brain (B), thymus (T), pancreas (P), and blood (BL), were collected. Subsequently, TMUV antigen, CD4, and CD8α molecule distribution in SP, LI, and B were analyzed by IHC, which was performed according to our previous study (25).

### Characterization of GoIFN Expression during TMUV Infections

In an *in vitro* study, GEFs were infected with 100 µL TMUV (contained 1,000 TCID_50_), and the control groups were treated with the same dose of PBS. At 12, 24, 36, and 48 h post-infection (hpi), the cells were harvested with 1 mL RNAiso Plus Reagent for RNA extraction, and goIFN (IFNα, IFNγ, and IFNλ) mRNA was detected by quantitative real-time PCR (RT-qPCR). Additionally, in the *in vivo* study, immune-associated tissues, such as SP, LI, B, T, P, and BL, from TMUV-infected goslings (1–4 dpi), were collected with 1 mL RNAiso Plus reagent for RNA extraction and goIFN mRNA was detected by RT-qPCR.

### Reporter Gene Assay

The pGL3-IFNβ-Luc expression plasmid was constructed with the sequence of the duck IFNβ promotor region (GeneBank accession number: KM032183.1). The commercialized pGL4-IRSE-Luc expression plasmid was purchased from Promega (Madison, WI, USA). Originally, GEFs were seeded onto a 48-well plate and transiently transfected with the pGL3-IFNβ-Luc (400 ng/well) or pGL4-IRSE-Luc (400 ng/well). Subsequently, cells were transfected with pRL-TK plasmid (40 ng/well) (Promega, Madison, WI, USA), which acted as an internal control to normalize transfection efficiency. 24 h later, cells were challenged with 100 µL TMUV (contained 1000 TCID_50_). At 12, 24, 36, and 48 hpi, the cells were harvested for luciferase assays. The luciferase activities were determined with a Dual-GloLuciferase Assay System (Promega) and normalized based on the Renilla luciferase activity.

### Antiviral Assay

The recombinant plasmids pcDNA3.1 (+)-goIFN-α, γ, and λ were transfected into BHK-21 cells. Cell lysates from BHK-21 cells were harvested at 24 h post-transfection and clarified by centrifugation at 500 × *g* for 10 min after freezing and thawing three times. Then, GEFs were incubated with 100 µL goIFN-α, γ, and λ. After 12 h of incubation, cells were infected with 400 µL TMUV (contained 1000 TCID50). At 36, 48, and 60 hpi, cells were collected for the detection of viral copies and viral titers. Samples (200 µL) were extracted with a nucleic acid extraction kit (Tiangen, Shanghai), and then TMUV copies were detected by RT-qPCR using the special primers based on the TMUV-E gene (shown in Table 1). Subsequently, TMUV titers were determined by an endpoint dilution assay in GEFs and the results were analyzed using the Reed–Muench method (TCID_50_).

**Table 1:** The list of primers sequences used for qPCR in this study.

Primer name	Nucleotide sequence (5′–3′)
goGAPDH-qPCR-F	CATTTTCCAGGAGCGTGACC
goGAPDH-qPCR-R	AGACACCAGTAGACTCCACA
goIFN-α-qPCR-F	CAGCACCACATCCACCAC
goIFN-α-qPCR-R	TACTTGTTGATGCCGAGGT
goIFN-γ-qPCR-F	TGAGCCAGATTGTTTCCC
goIFN-γ-qPCR-R	CAGGTCCACGAGGTCTTT
goIFN-λ-qPCR-F	GAGCTCTCGGTGCCCGACC
goIFN-λ-qPCR-R	CTCAGCGGCCACGCAGCCT
goMx-qPCR-F	TTCACAGCAATGGAAAGGGA
goMx-qPCR-R	ATTAGTGTCGGGTCTGGGA
goOASL-qPCR-F	CAGCGTGTGGTGGTTCTC
goOASL-qPCR-R	AACCAGACGATGACATACAC
goViperin-qPCR-F	CGTTAGCAACGGCAGCCTGAT
goViperin-qPCR-R	CATACTCGCGGCACCACTGT
goTRIM25-qPCR-F	CCACCACCCTCAGCGTTTC
goTRIM25-qPCR-R	GCCATAGCAGATGCCAAT
goIFITM5-qPCR-F	CTACCCACGGGAGGACTA
goIFITM5-qPCR-R	AAGCCAAGGCAGCAGAAG
goRIG-I-qPCR-F	AGCACCTGACAGCCAAAT
goRIG-I-qPCR-R	AGTGCGAGTCTGTGGGTT
goMDA5-qPCR-F	TGCTGTAGTGGAGGATTTG
goMDA5-qPCR-R	CTGCTCTGTCCCAGGTTT
goIRF7-qPCR-F	ACCCGCCTGAAGAAGTGC
goIRF7-qPCR-R	GAGAAGCACTGCCCGAAG
goNF-kB-qPCR-F	TCCCAATGCCTCCAACTT
goNF-kB-qPCR-R	AGCCTTCCCACATACCAC
goSTAT1-qPCR-F	CAGAGCCTATGGATTTGGAT
goSTAT1-qPCR-R	CCACCATCCGAGACACCT
goJAK2-qPCR-F	AGCACCTTAGGGACTTCG
goJAK2-qPCR-R	CTTGTGGTCCAGTCTTTCC
goSOCS1-qPCR-F	GCAGGCACAGAGCAACACG
goSOCS1-qPCR-R	CCCTCGGGCTCAGACTTCA
goSOCS3-qPCR-F	GGTCACCCACAGCAAGTT
goSOCS3-qPCR-R	TGAGCGTGAAGAAGTGCC
goUSP18-qPCR-F	GACAGAACAGCAGAGCCAAGC
goUSP18-qPCR-R	TCCCACGATACCTGACAAACG
TMUV-qPCR-F	CGCTGAGATGGAGGATTATGG
TMUV-qPCR-R	TCTCACTCGGAAGGACATAT

### RNA Interference

Small interfering RNAs (siRNAs) targeting goMx and goOASL were chemically synthesized by GenePharma (China). The target sequences for the knockdown of goMx and goOASL are presented in Table 2. The target sequences were inserted into a pGPU6 plasmid to generate pGPU6-shgoMx (shMx_120_, shMx_699_, and shM_1146_) and pGPU6-shgoOASL (shOASL_549_, shOASL_1113_, and shOASL_1476_). A non-targeting shRNA (NC-shRNA) was synthesized as a negative control. The efficiencies of the siRNA were measured by RT-qPCR and Western blot.

**Table 2:** The list of primers sequences used for RNAi assay in this study.

Primer name	Target sequense (5′–3′)
pGPU6-shMx_120_	GCCACCAGATTTGGATGATCA
pGPU6-shMx_699_	GCCACAAGACATTGGAGAACA
pGPU6-shMx_1146_	GCCTACAATCGAGAACCAAAT
pGPU6-shOASL_549_	GGGAATTCTCCACCTGCTTCA
pGPU6-shOASL_1113_	GGCAGATGAAGGAAATGATCG
pGPU6-shOASL_1476_	GCACTATCTTCCTGCTTCTGC

### Sample Preparation and cDNA Library Construction

PBMCs were stimulated with recombinant goIFN-α, γ, and λ for 3 h, and those treated with cell lysates from pcDNA3.1 (+)-transfected BHK-21 cells were considered as control group. Cells were harvested with 1 mL RNAiso Plus reagent (Takara Bio, Otsu, Japan) for RNA extraction.

Obtained RNA quantity and quality were evaluated using NanoDrop, Qubit, and Agilent 2100 Bioanalyzer (Agilent Technologies, Palo Alto, CA, USA). The RNA integrity number and purity (28S/18S) of all samples conformed to the requirements of a standard procedure (21). The mRNA of all qualified samples was enriched using oligo (dT) magnetic beads, and fragmented mRNAs were reverse transcribed into single strand cDNA using random hexamer primers. The cDNAs were converted into double strand cDNAs with DNA polymerase I and purified using AMPure XP beads. After qualification and quantification using an Agilent 2100 Bioanalyzer and ABI Step One Plus Real Time PCR System, the libraries were sequenced with Illumina HiSeq™ 2000. To obtain high-quality clean reads for assembly, the raw reads were filtered through the NGS QC TOOLKIT by removing low quality reads. All the clean reads were pooled and assembled using the Trinity *de novo* assembly program (29). RNA-seq data can be accessed under GSE101404.

### Analysis of Differentially Expressed Genes (DEGs)

Comparisons between IFN treatment groups and the control group were performed, and DEGs were analyzed using the DESeq R package (30), a model based on the negative binomial distribution. For the statistical analysis, all read counts were normalized by calculating the FPKM value (31), and further, the FPKM + 1 values were log2 transformed and the means of expression (in log2 FPKMs) were used for further analysis. An *P*-value <0.05, which was adjusted by the false discovery rate, was defined as the threshold of DEGs, and log2-fold change > 0 (or <0) was defined as upregulated (downregulated genes). A Venn diagram was constructed to show the numbers of DEGs that were either specific or commonly induced among the three IFN treatment groups. Volcano and heat map hierarchical clustering analysis were performed to obtain the distribution and expression pattern of DEFs, respectively. Additionally, DEGs were assigned functional annotations using KEGG pathway enrichment to find immune-related genes (IRGs) for further analysis.

### The Validation of RNA-Seq Data by RT-qPCR

According to the results of DEG analysis, we selected 17 IFN-responsive genes from the RNA-seq data for validation by RT-qPCR. In brief, total RNA was extracted using 1 mL RNAiso Plus reagent, and RT-PCR was performed on each sample using a 5X All-In-One RT MasterMix Reagent Kit in accordance with the manufacturer’s instructions (Applied Biological Materials, Richmond, BC, Canada). Finally, the cDNA was stored at −80°C until use. Then, the expression of candidate genes was detected by RT-qPCR performed using the Bio-Rad CFX96 Real-Time Detection System (Bio-Rad, USA), and threshold cycle (Ct) values were normalized to the housekeeping genes duβ-actin or goGAPDH. The relative expression levels of each target gene were calculated with the comparative Ct (2^−ΔΔCt^) method. Finally, primers were designed using the cDNA in the sequence database (Table 1).

### Data Statistics

The statistical analyses were performed with GraphPad Prism 5 (GraphPad Software Inc., San Diego, CA, USA). The differences between values were evaluated by Student’s *t*-test. *P* < 0.05 was considered statistically significant, and all values were expressed as the mean ± SEM.

## Results

### Effects of TMUV in GoIFN Expression *In Vivo* and *In Vitro*

**Figure 1:**
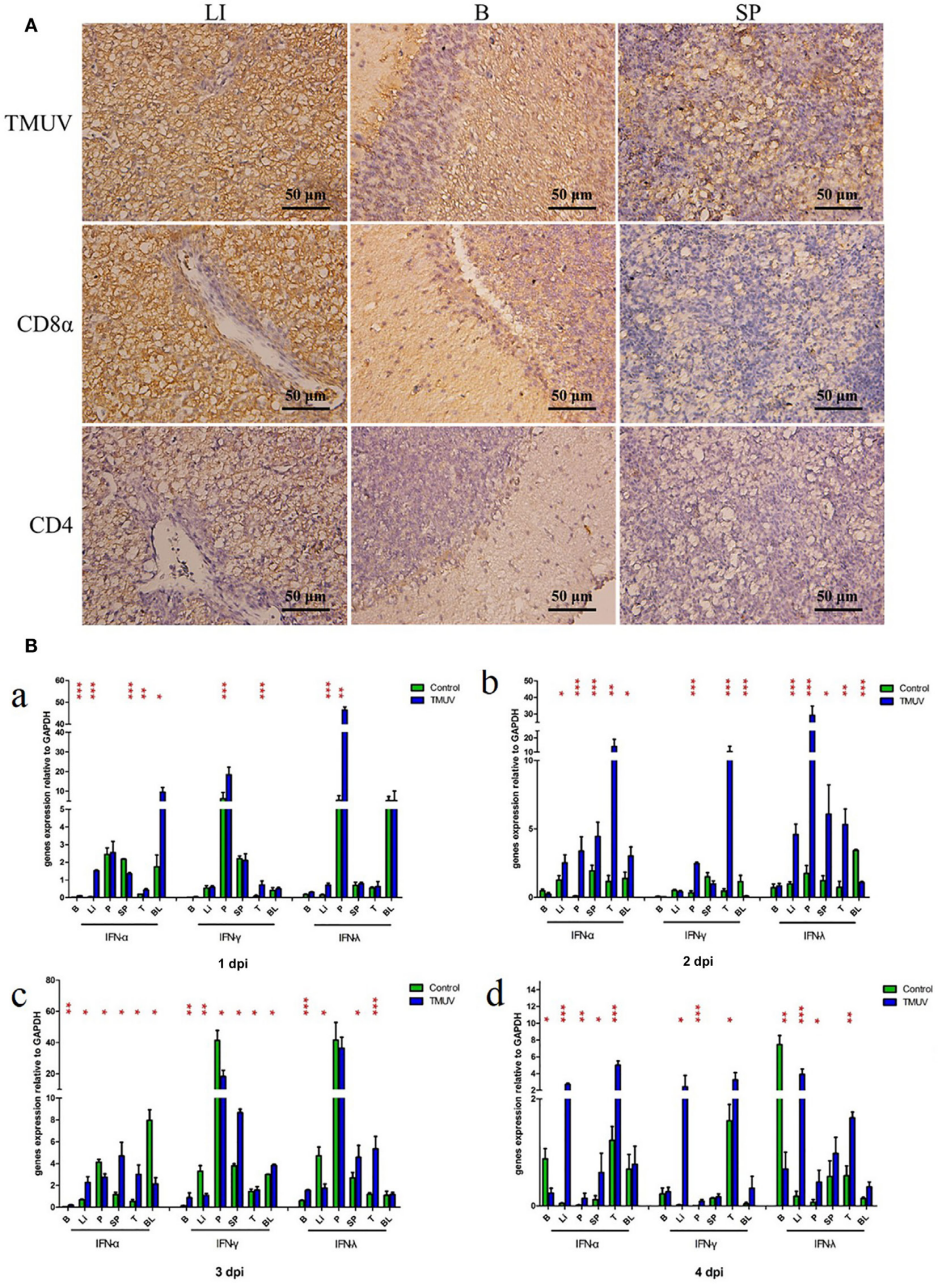
The distribution of viral antigen, CD8α and CD4 as well as the transcription levels of goIFNs in TMUV-infected goslings. **(A)** The distribution of TMUV antigen and CD8α and CD4 molecules in the liver (LI), brain **(B)**, and spleen (SP) were measured by IHC assay. The dark brown represents positive signaling for TMUV, CD4, and CD8α antigen using immunohistochemical staining, and sections were counterstained with hematoxylin (blue). Rabbit polyclonal antibody against TUMV E protein, mouse anti-duck monoclonal CD4 antibodies (1:100) and mouse anti-goose polyclonal CD8α antibodies (1:100) were used as the primary antibody, while the goat anti-mouse and goat anti-rabbit antibodies were used as the secondary antibody. **(B)** The mRNA levels of goIFNs in the immune-associated tissues of 1–4 post-infected gosling, such as the spleen (SP), liver (LI), brain (B), thymus (T), pancreas (P), and blood (BL), were detected by quantitative real-time PCR. All results were normalized to GAPDH. Data are represented as the mean ± SEM (*n* = 4). Significant differences from the mock groups are indicated by *P < 0.05, **P < 0.01, and ***P < 0.001.

**Figure 2:**
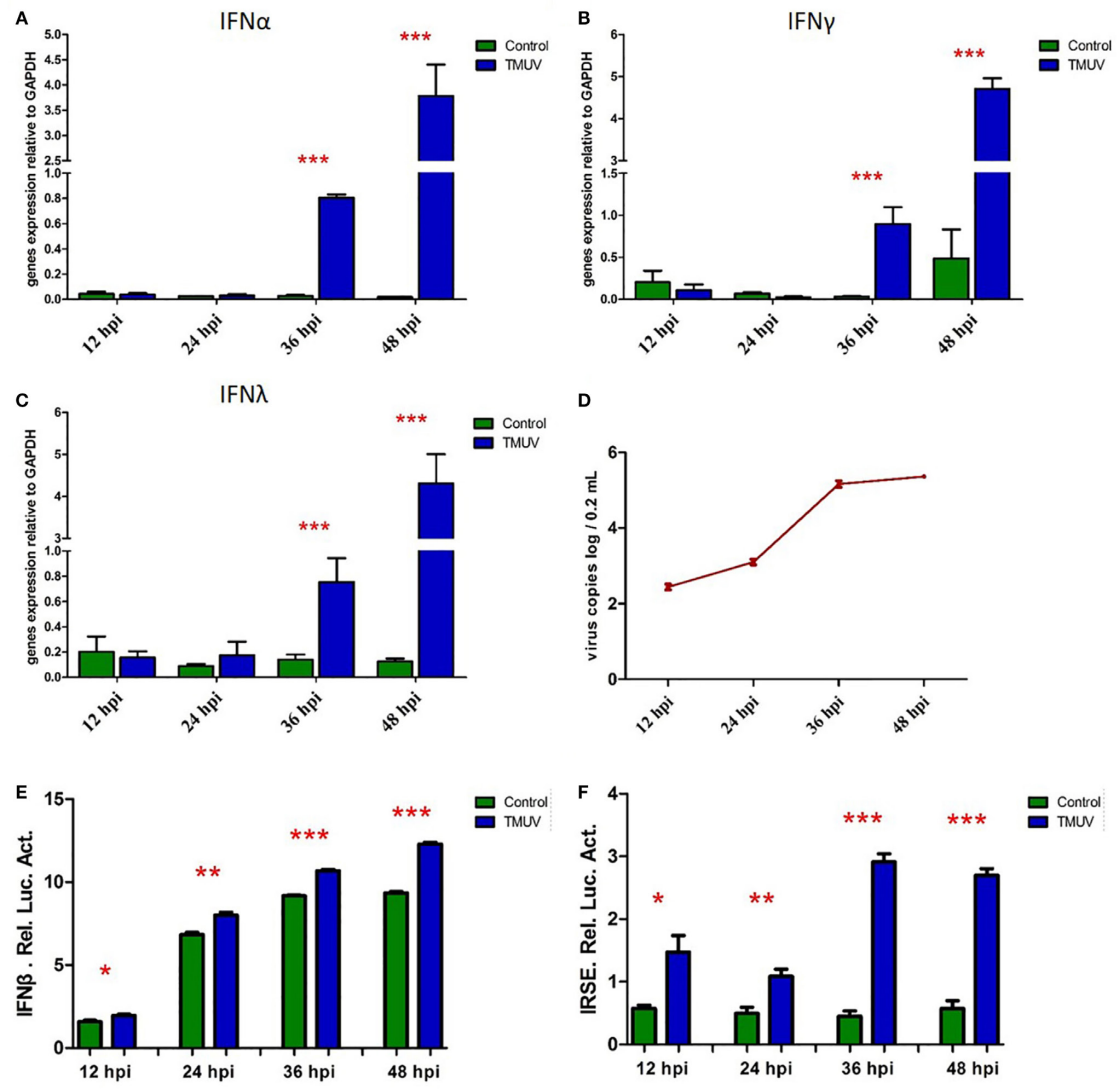
The transcription levels of goIFNs in TMUV-infected goose embryo fibroblasts (GEFs). **(A–C)** GEFs were treated with 100 µL TMUV (1,000 TCID_50_), cells were harvested at 12, 24, 36, and 48 hours post-infection. Then, the expression level of goIFNs mRNA was detected by quantitative real-time PCR. All results were normalized to GAPDH. Data are represented as the mean ± SEM (*n* = 4). Significant differences from the mock groups are indicated by *P < 0.05, **P < 0.01, and ***P < 0.001. **(D)** Virus copy detection of TMUV-infected GEFs at 12, 24, 36, and 48 h. **(E,F)** Luciferase activities (IFNβ-luc and IRSE-luc) were determined with a Dual-Glo^®^Luciferase Assay System (Promega) and normalized based on the Renilla luciferase activities. All luciferase reporter assays were repeated three times.

To determine the production of goIFNs in response to TMUV challenge, we infected the GEFs and goslings with duck TMUV. In the *in vivo* study, 3-day-old goslings were infected with TMUV (i. m 500 µL). As shown in Figure 1A, the TMUV antigen was markedly distributed in the SP, LI, and B, which was highly connected with the distribution of the CD8α molecule. We also found that goIFNs were differentially upregulated in all selected tissues during TMUV infection (1–4 dpi), with especially high expression in immune-related tissues, such as LI, SP, and T (Figure 1B, a–d). Notably, significant upregulation of goIFNα was shown in LI and T at all time points, and goIFNγ was always markedly upregulated in T, while the expression level of goIFNλ was almost increased in LI, SP, and T by TMUV. Meanwhile, in the *in vitro* study, continuous upregulation of goIFNs was detected in GEFs with increasing TMUV infection time (12, 24, 36, and 48 hpi) (Figures 2A–D). Moreover, this upregulation was also detected by reporter assays, and TMUV infection triggered the activation of the IFNβ promoters and IRSE in GEFs at 12, 24, 36, and 48 hpi (Figures 2E,F). Taken together, these results provide evidence that TMUV infection strongly induces the transcription of goIFNs both *in vivo* and *in vitro*, which means that goIFNs play an important role in TMUV defense.

### Antiviral Activity of GoIFNs against TMUV Infection

**Figure 3:**
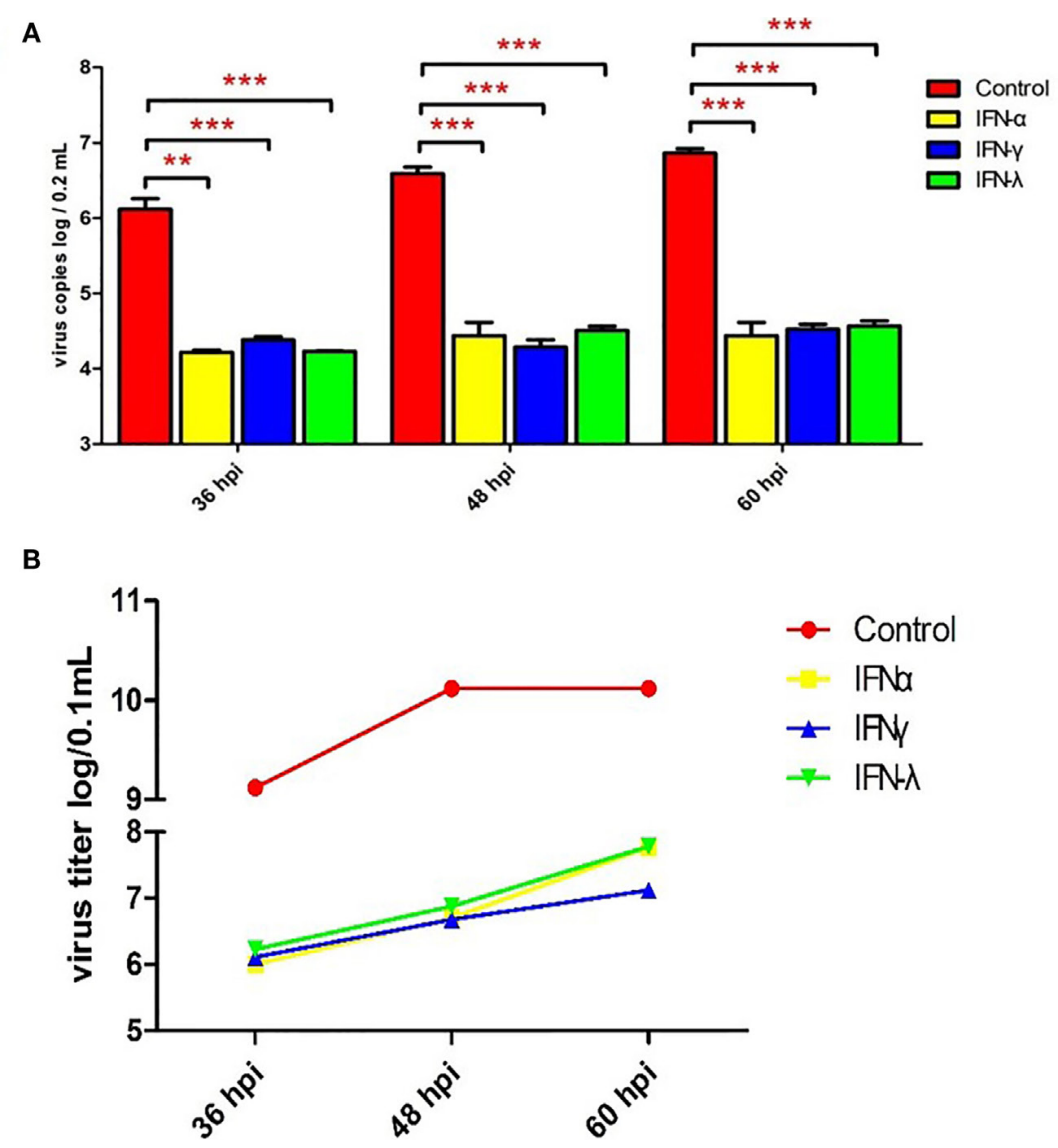
Pre-treating goose embryo fibroblasts (GEFs) with goIFNs reduced the replication of TMUV. GEFs were pre-treated with 100 µL goIFNα, goIFNγ, and goIFNλ (from goIFNs plasmids transfected BHK-21 cells for 12 h). The control group was treated with 100 µL of the cell lysates from pcDNA3.1 (+)-transfected BHK-21 cells. Subsequently, the cells were infected with 400 µL TMUV (contained 1000 TCID_50_). Cells were harvested at 36, 48, and 60 hpi for the detection of virus copies **(A)** and titers **(B)**. Four experimental replicates were performed, and all of the results showed the mean ± SEM. Significant differences between the mock groups are indicated by *P < 0.05, **P < 0.01, and ***P < 0.001.

To explore the effect of goIFNs against TMUV replication, an antiviral activity assay was performed. As Figure S1 in Supplementary Material shows, goIFNs did not show any cytotoxic effect on GEFs. Both viral copies and titers of TMUV were significantly decreased compared with the control group (Figure 3). Therefore, it is believed that pre-treatment with goIFNs prior to infection inhibits the replication of TMUV in GEFs.

### Effects of GoIFNs on ISG Expression in Goose PBMCs

**Figure 4:**
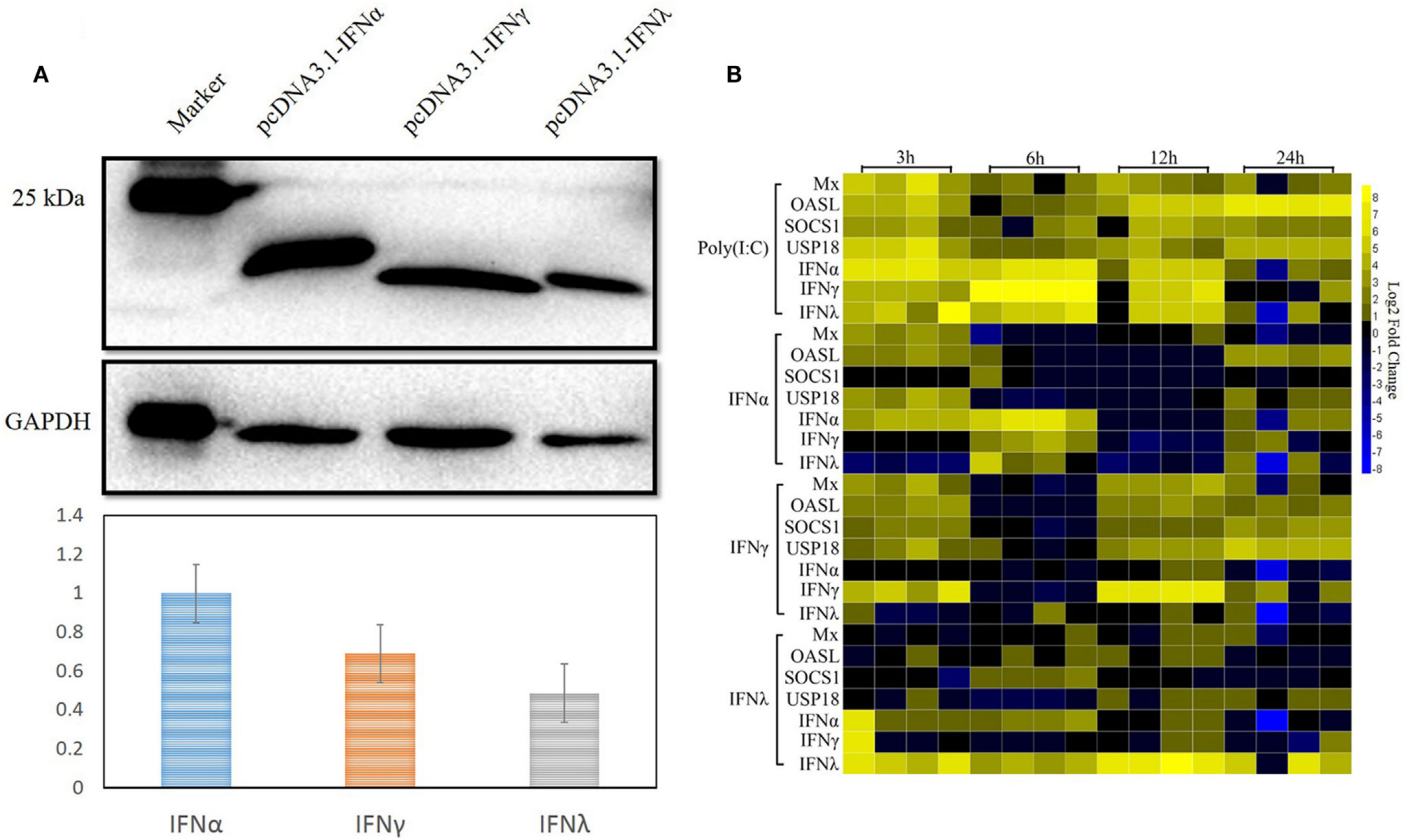
Interferon-stimulated genes were induced by goIFNs in PBMCs. **(A)** Western blot analysis of goIFNα, goIFNγ, and goIFNλ expressed in the transfected BHK-21 cells. BHK-21 cells were transfected with pcDNA3.1 (+) vector or pcDNA3.1 (+)-goIFNs (α, γ, and λ) recombinant plasmid for 24 h. Cell lysates were measured by Western blot. A monoclonal antibody against his-tag (1:10,000 dilution) and an HRP-labeled goat anti-mouse IgG antibody (1:2,000 dilution) were used as the primary antibody and secondary antibody, respectively. The gray intensity was analyzed by Image J software. **(B)** Heat map analysis of goIFN-regulated genes in PBMCs. PBMCs were treated with goIFNα, goIFNγ, and goIFNλ, while the poly (I:C)-treated group was considered a positive control. Cells were collected for detection by quantitative real-time PCR. Each column represented a time point (3, 6, 12, and 24 h), and each row represented a unigene (goIFNα, goIFNγ, goIFNλ, goMx, goOASL, goSOCS-1, and goUSP18). The differences in the expression level were shown in distinct colors. Yellow indicated a high expression level, while blue indicated a low expression level.

As already reported by others, IFNs can induce the expression of a large number of ISGs, leading to the induction of the antiviral state to prevent pathogen invasion (32–35). In our previous study, both goIFNα and goIFNγ could trigger the expression of downstream ISGs in GEFs and DEFs, which inhibited DNA virus (duck plague virus) replication in DEFs (36). Here, we assumed that the pre-treatment of GEFs with goIFNs induced the expression of ISGs that protect cells from TMUV infection. Therefore, it is necessary to expand our understanding of the global gene expression profile of goose PBMCs after stimulation with goIFNs. Initially, goIFNα, goIFNγ, and goIFNλ concentrations were assessed based on the results of Western blotting using ImageJ software with GAPDH as a loading control protein. Our finding showed that the protein level of 50 µL of IFNα equal to 70 µL of goIFNγ and 100 µL of goIFNλ (Figure 4A). Therefore, PBMCs were stimulated with equal levels of goIFN-α, γ, and λ. Under the same experimental conditions, Poly (I:C) was chosen as a positive control, and cell lysates from pcDNA3.1 (+)-transfected BHK-21 cells were considered as the experimental control group. At 3, 6, 12, and 24 hpi, cells were harvested for the detection of IFN (IFNα, IFNγ, IFNγ) and ISG (Mx, OASL, SOCS-1, and USP18) mRNAs by RT-qPCR. The highest transcription level of ISGs was shown in the first time point (3 h) following the treatment of IFNs in PBMC (Figure 4B). Therefore, 3-h samples of goIFNs stimulated cells were further subjected to RNA-seq by Illumina HiSeq™ 2000 (Novegene, Beijing).

### Summary of Transcriptome Data

Mock and IFN-stimulated RNA samples were collected for deep sequencing using Illumina HiSeq 2000. The statistics of twelve libraries are shown in Table 3. Briefly, more than 60 million raw reads were generated for each library, with an error rate of 0.01–0.02%. After trimming, more than 95% of the raw reads were clean reads for each library. Additionally, at least 60% of the paired-end reads mapped to the goose (*Anser*) genome, with a phred quality score (Q30) above 90%. These data suggested that the accuracy and quality of the sequencing data were sufficient for further annotation and expression analysis.

**Table 3:** Summary for RNA-Seq datasets of goIFNs.

Sample name	Raw reads	Clean reads	Error rate (%)	Q30 (%)	Mapped reads (%)
Mock_1	76534582	73335882	0.02	90.52	47435639 (64.68)
Mock_2	70538388	67739916	0.02	90.78	44159188 (65.19)
Mock_3	63584470	60208238	0.02	89.16	38040357 (63.18)
IFNα_1	63791120	61554582	0.02	91.94	41254093 (67.02)
IFNα_2	69247654	66834280	0.02	89.36	44303391 (66.29)
IFNα_3	68840982	66885614	0.02	92.87	44487544 (66.51)
IFNγ_1	83258992	80967152	0.02	93.10	53198810 (65.7)
IFNγ_2	75352158	73019346	0.02	92.70	46960495 (64.31)
IFNγ_3	63143564	61301556	0.01	93.23	39527705 (64.48)
IFNλ_1	79769402	77414546	0.01	93.37	50608450 (65.37)
IFNλ_2	69873162	67879200	0.01	93.44	43696450 (64.37)
IFNλ_3	70912982	68112134	0.02	92.89	42781011 (62.81)

### Global Profiling of Gene Expression in Response to GoIFNs in PBMCs

**Figure 5:**
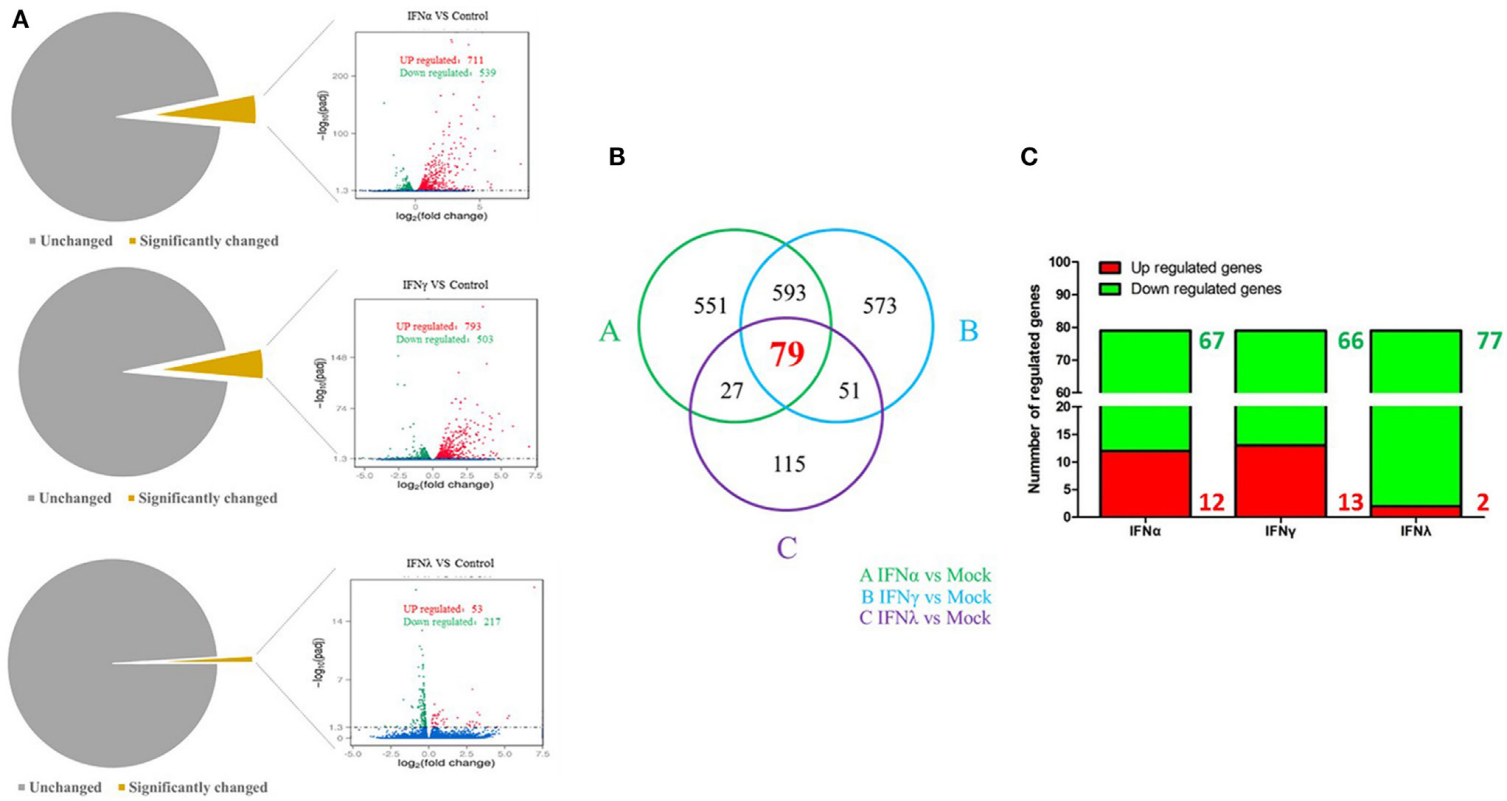
The analysis of differentially expressed genes (DEGs) based on transcriptome analysis. **(A)** Volcano plot analysis of DEGs. Each plot represents a unigene. Red indicates upregulated genes, while green indicates downregulated genes. **(B)** Venn diagram analysis of DEGs. Red highlights indicate the numbers of genes commonly regulated by goIFNα, goIFNγ, and goIFNλ. **(C)** The proportion of up- and downregulated genes commonly regulated by goIFNs. Likewise, red indicates upregulated genes, while green indicates downregulated genes.

To uncover the innate immune response to goIFNs, we first set out to identify the response of DEGs to goIFNs by pairwise comparisons to mock-treated controls. Overall, based on the criteria that |fold-change| ≥ 2 and *P*-value ≤ 0.01, we observed 1250 DEGs (4.5% of total genes) and 1296 DEGs (4.6% of total genes) in goIFNα vs. controls and goIFNγ vs. controls, respectively (Figure 5A). In goIFNα vs. control data, 711 genes were upregulated and 539 were downregulated, while in goIFNγ vs. control data, 793 genes were upregulated and 503 were downregulated. Notably, lower DEGs (1% of total genes) were identified in goIFNλ vs. control; among these, 53 were upregulated and 217 were downregulated (Figure 5A). Moreover, a total of 79 co-expressed genes were commonly regulated by all goIFNs (Figure 5B). Of these 79 common genes, 12 were upregulated and 67 were downregulated by goIFNα, 13 were upregulated and 66 were downregulated by goIFNγ, while 2 were upregulated and 77 were downregulated by goIFNλ (Figure 5C). These observations indicated that the goIFNα treatment group displayed similar gene expression to the goIFNγ-treated group, while treatment with equal levels of goIFNλ resulted in mild changes in subsequent gene expression.

### Identification and Confirmation of IRGs

**Figure 6:**
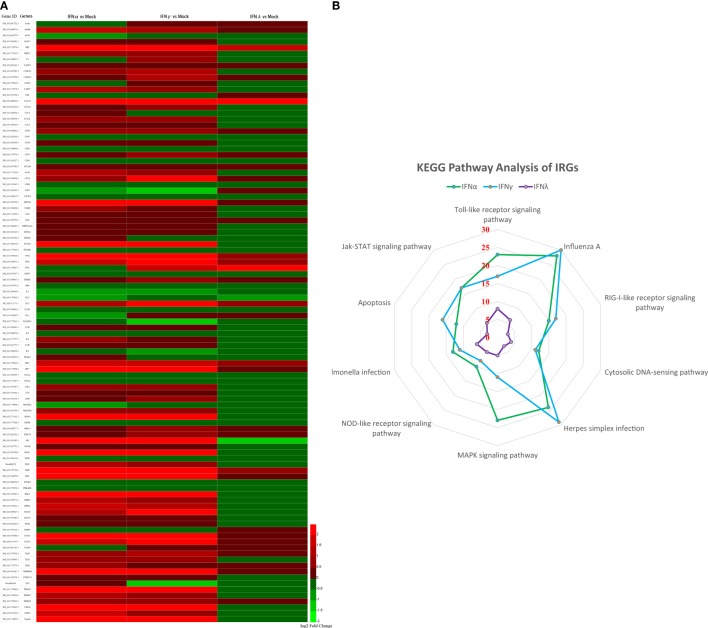
Statistical analysis of immune-related genes (IRGs) based on transcriptome analysis. **(A)** Expression pattern of IRGs. Each column represents a sample, and each row represents a unigene. Red indicates upregulated genes, while green indicates downregulated genes. **(B)** KEGG pathway analysis of IRGs. IRGs of goIFNs are mainly involved in the toll-like receptor signaling pathway, Jak-STAT signaling pathway, Influenza A infection pathway and herpes simplex infection pathway.

**Figure 7:**
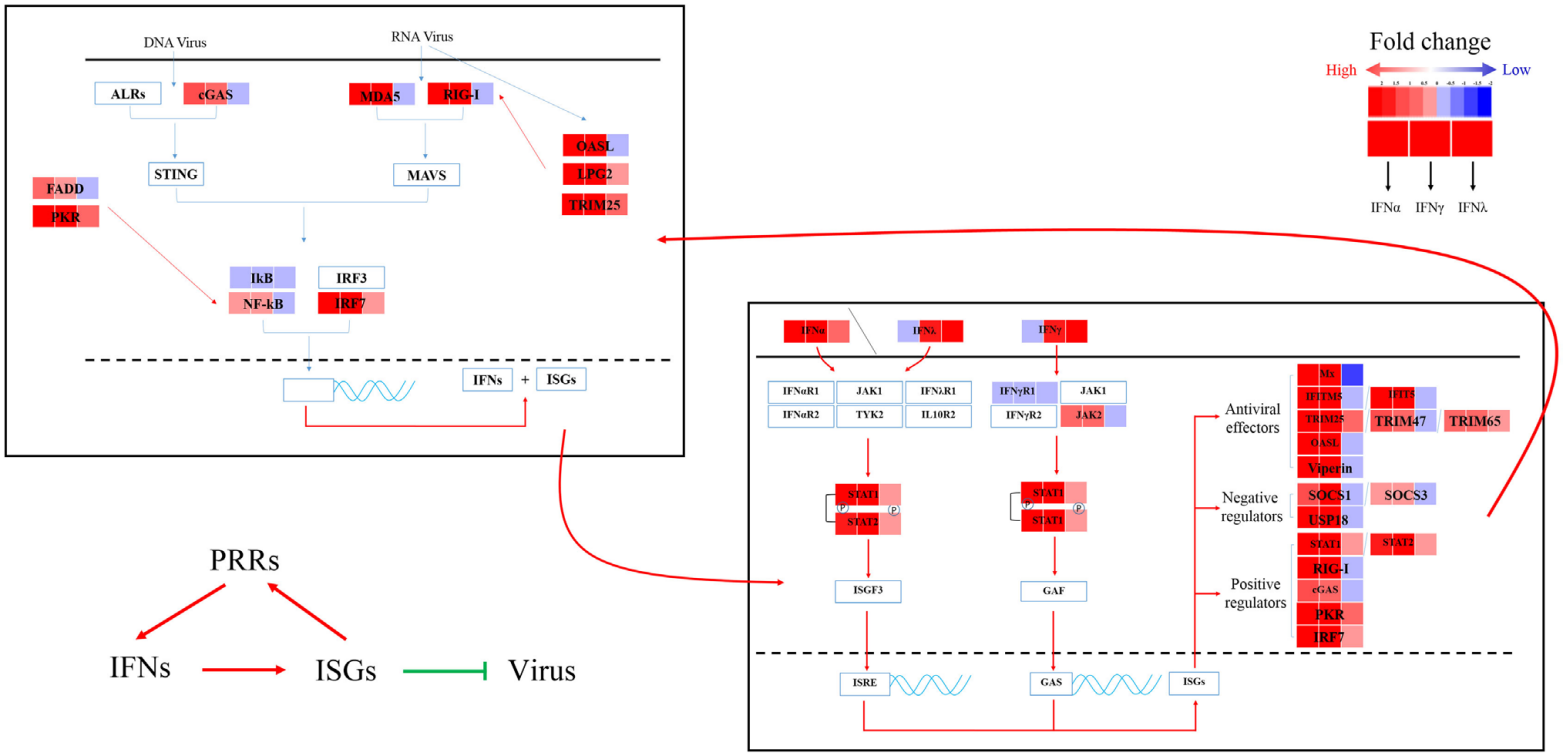
Sketch map of the goose antiviral response against avian TMUV infection in an IFN-dependent way. Red indicates upregulated genes, while blue indicates downregulated genes.

To further understand the antiviral mechanism of goIFNs, the IRGs among the identified DEGs were listed. A total of 101 IRGs were defined as ISGs (Figure 6; Table S1 in Supplementary Material), including IFNs, positive regulators (RIG-I, cGAS, PKR), negative regulators (SOCS-1, USP18) and antiviral effectors (Mx, OASL, IFITM5, TRIM25). The top 10 IRGs are presented in Tables S2–4 in Supplementary Material. KEGG analysis showed that the IRGs are mainly involved in the toll-like receptor signaling pathway, JAK-STAT signaling pathway, influenza virus infection pathway and herpes simplex infection pathway. Additionally, we obtained specific regulated genes through the pairwise comparison of goIFNα, goIFNγ, and goIFNλ (Figures S2A–C in Supplementary Material). These findings provided evidence that each type of goIFN induces some unique ISGs, and all goIFNs were required to confer the expression of IRGs for innate immune protection and the restriction of viral infection (Figure 7).

**Figure 8:**
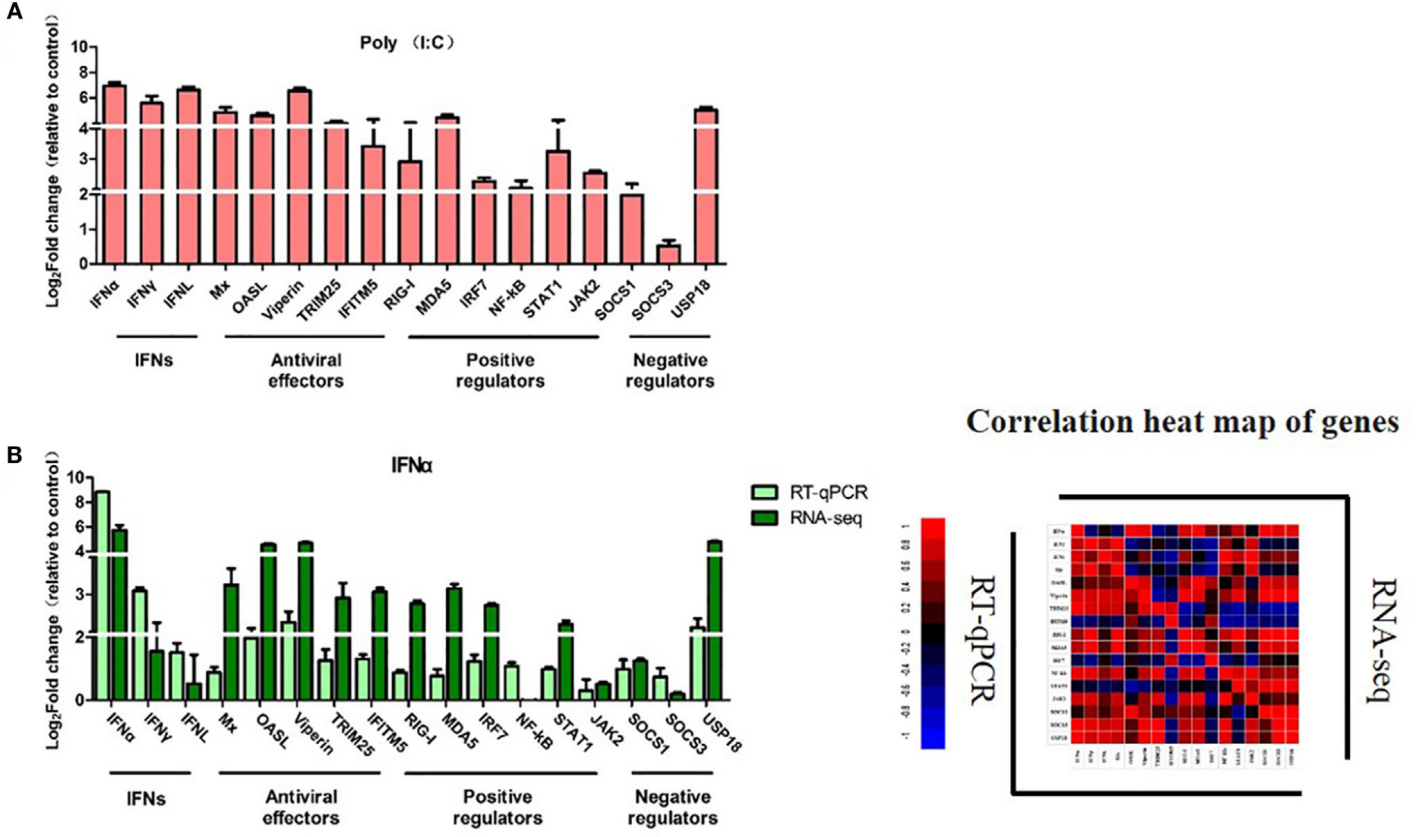

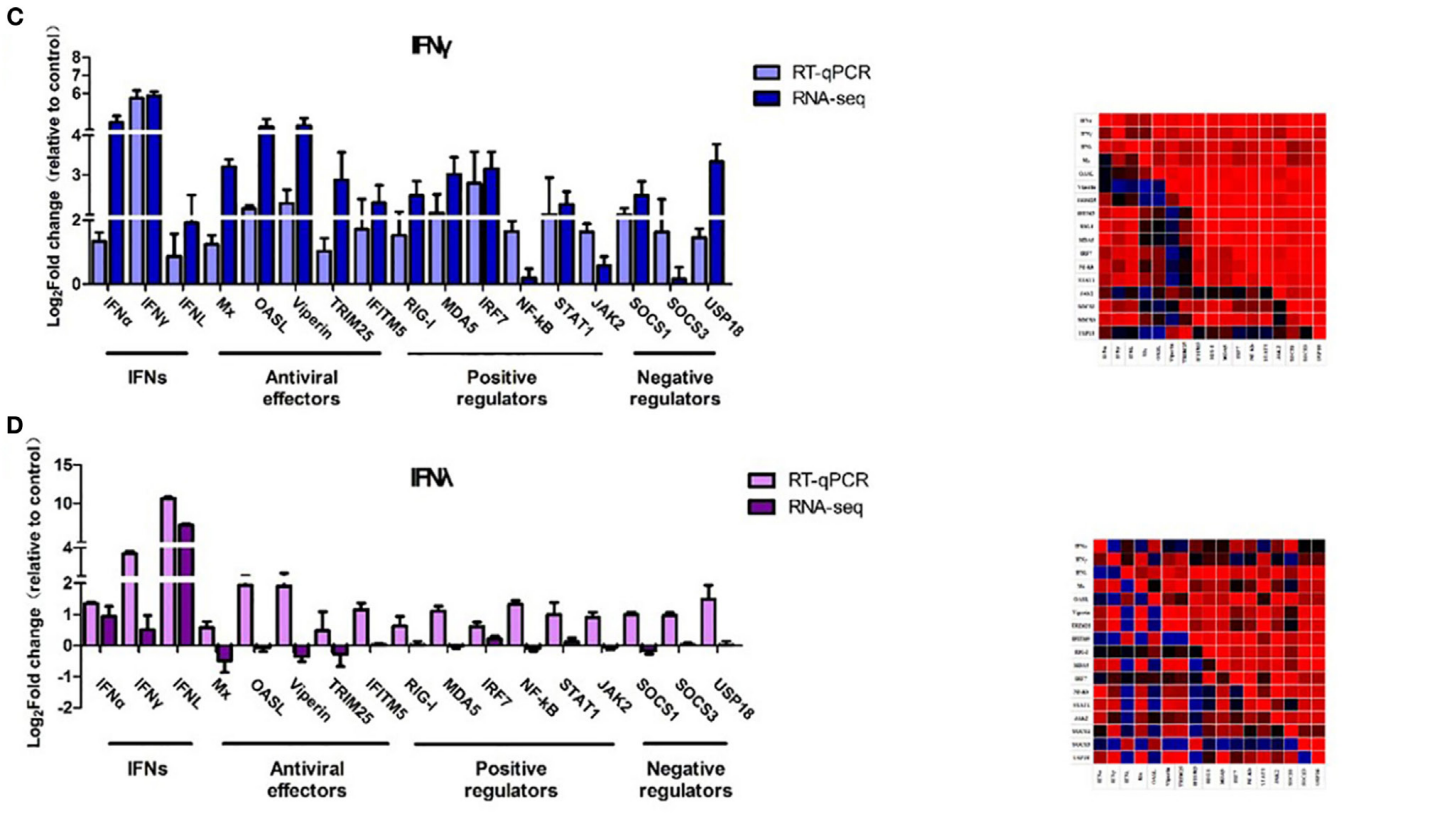
Quantitative Real-time PCR (RT-qPCR) validation of 17 candidate genes. Goose PBMCs were treated with **(A)** Poly(I:C) (30 ng/mL) and **(B)** goIFNα, **(C)** goIFNγ, **(D)** goIFNλ for 3 h. The mRNA expression level of candidate genes was detected by RT-qPCR. All results were normalized to GAPDH. Correlation analysis of candidate genes from the results of RT-qPCR and RNA-seq was performed by SPSS software. All data were represented as the mean ± SEM (*n* = 4). Significant differences from the mock groups are indicated by *P < 0.05, **P < 0.01, and ***P < 0.001.

To validate the RNA-Seq results, three types of goIFNs, five antiviral effectors (Mx, OASL, Viperin, TRUM25, and IFITM5), six positive regulators (RIG-I, MDA5, IRF7, NF-kB, STAT1, and JAK2) and three negative regulators (SOCS-1, SOCS-3, and USP18) were chosen for RT-qPCR analysis (Figure 8). Goose PBMCs were treated with goIFNs for 3 h. Meanwhile, PBMCs were treated with pCDNA3.1(+) and Poly(I:C) (30 ng/mL) for 3 h as negative and positive control groups. According to the RT-qPCR analysis results, the expression patterns of most of the genes induced by goIFNα and goIFNγ were highly consistent with those found in the RNA-Seq analysis (Figure 8). However, the RT-qPCR results suggested that a higher expression level of goIFNs induced more genes than RNA-seq (Figure 8), indicating the RNA-seq method is reliable in DEG identification. Moreover, all antiviral effectors (goMx, goOASL, goViperin, goTRIM25, and goIFITM5) were significantly upregulated by goIFNs. As the classical antiviral genes, goMx and goOASL were selected to confirm the roles in the goIFN-induced antiviral immune response.

### GoMx and GoOASL Play Key Roles in the Antiviral Effects of GoIFNs

**Figure 9:**
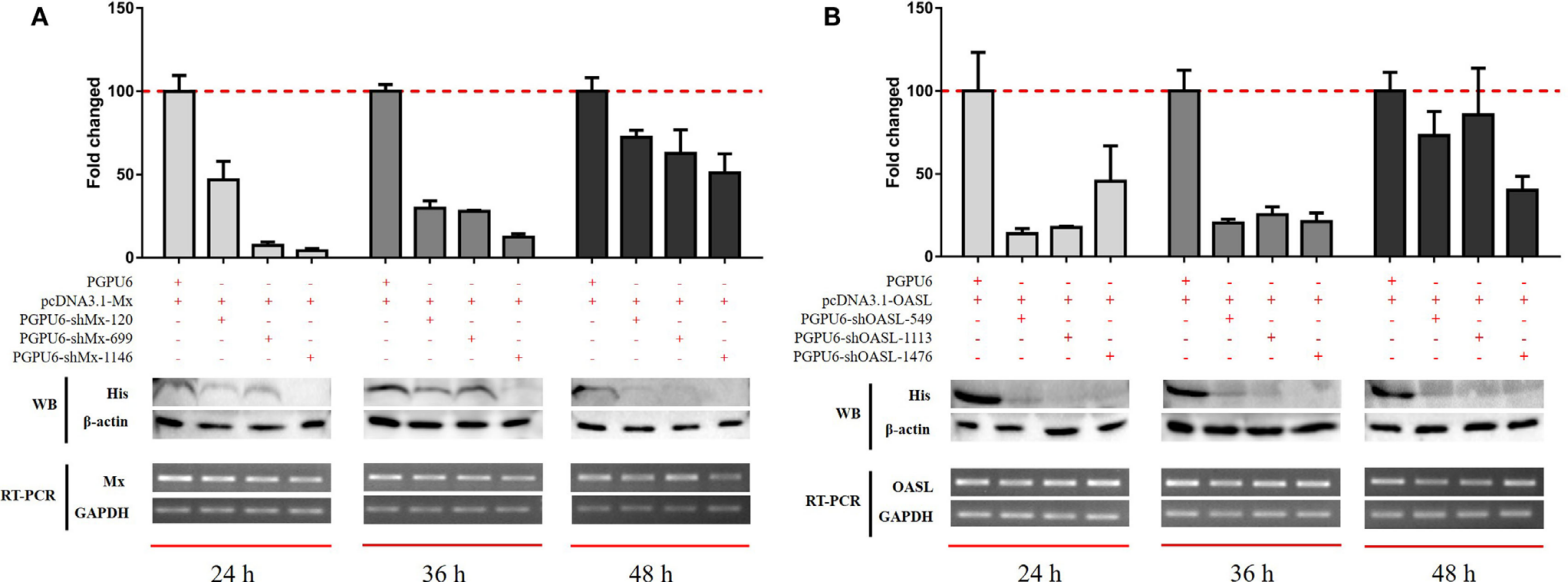
Optimum silencing efficiency of **(A)** sh-goMx and **(B)** sh-goOASL. Goose embryo fibroblasts (GEFs) were transfected with pcDNA3.1-goMx and pcDNA3.1-goOASL plasmids, while pcDNA3.1-vector transfected cells were considered as a control group. Meanwhile, cells were cotransfected with sh-goMx (120, 699, 1146) or sh-goOASL (549, 1113, 1476) and cotransfected with non-targeting small RNA as the negative control shRNA. At 24, 36, and 48 h post-transfection, cells were harvested for quantitative real-time PCR and Western blot analysis. All results were normalized to GAPDH and represented as the mean ± SEM (*n* = 4).

**Figure 10:**
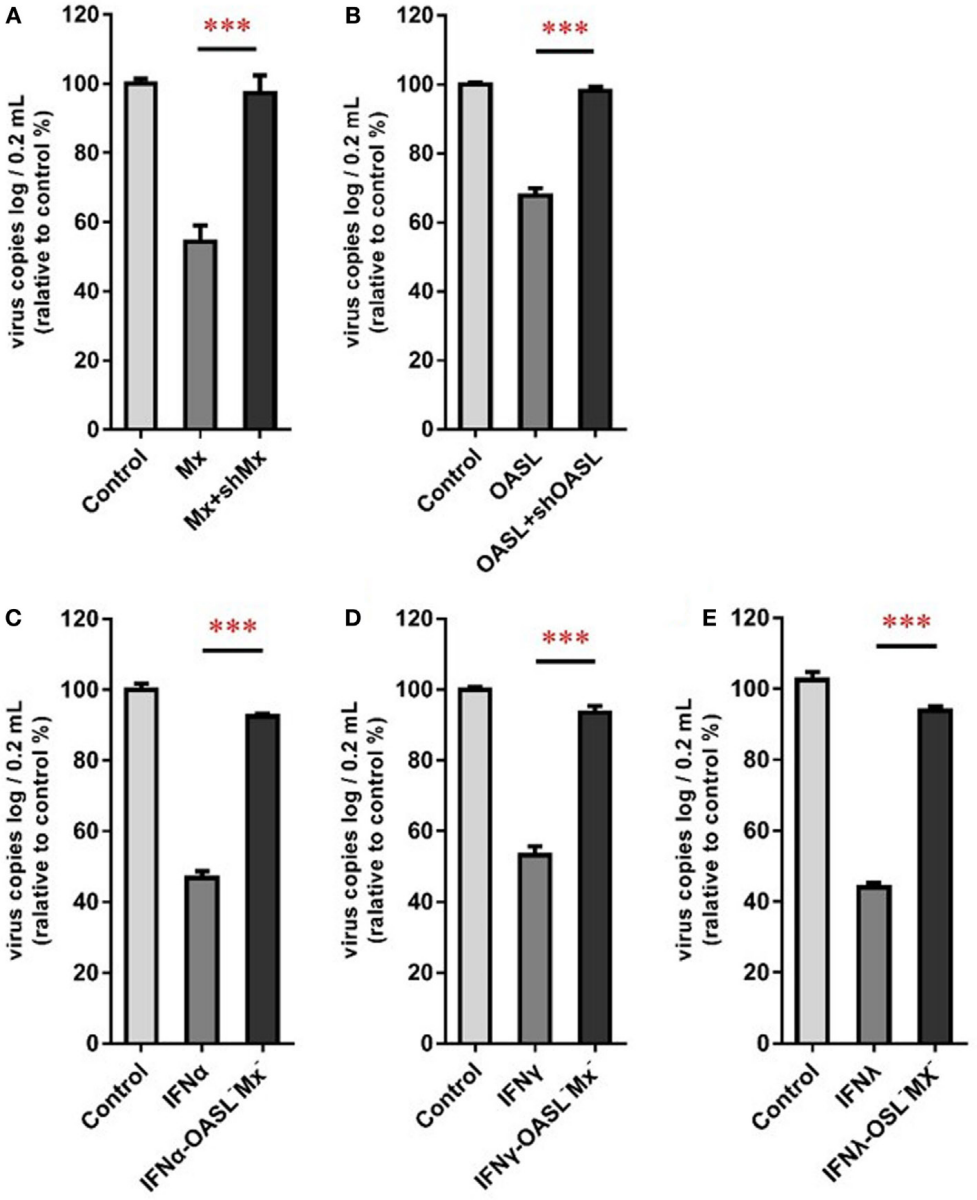
goMx and goOASL play a key role in goIFNs dependent antiviral effects. **(A,B)** GEFs were co-transfected with the pCDNA3.1-vector or goMx and goOASL plasmid, together with shMx_1146_, shOASL_549_, and NC-shRNA. After 24 h transfection, cells were infected with TMUV for 36 h, the virus copies were detected by quantitative real-time PCR (RT-qPCR); **(C–E)** GEFs were pretreated with goIFNs and subsequently transfected with shMx1146 and shOASL_549_. At 24 h post-transfection, cells were challenged with TMUV and virus copies were detected by RT-qPCR at 36 h. All data were represented as the mean ± SEM (*n* = 4). Significant differences from the mock groups are indicated by *P < 0.05, **P < 0.01, and ***P < 0.001.

To confirm the roles of goMx and goOASL in the goIFN-induced antiviral immune response, overexpression and knockdown by RNAi assay were performed. Initially, we found that cells transfected with shMx_1146_ and shOASL_549_ for 24 h were most efficient in knocking down goMx and goOASL expression, respectively (Figure 9). Then, cells were co-transfected with the pcDNA3.1-vector or pcDNA3.1-goMx and pcDNA3.1-goOASL plasmid, together with shMx_1146_ or shOASL_549_ and NC-shRNA. After 24 h transfection, cells were infected with TMUV for 36 h, and the viral copies were measured. RT-qPCR analysis showed that the overexpression of goMx or goOASL obviously reduced the TMUV replication, while the antiviral effect was reduced by the silencing of goMx and goOASL expression at 36 hpi, respectively (Figures 10A,B). These results indicated that goMx and goOASL conferred substantial protection against TMUV infection. Therefore, to confirm whether goMx and goOASL were associated with the goIFN-mediated antiviral effect, we pre-treated GEFs with goIFNs and subsequently transfected them with shMx_1146_ and shOASL_549_. At 24 h post-transfection, cells were challenged with TMUV, and viral copies were detected by RT-qPCR at 36 hpi. The results demonstrated that knockdown of goMx and goOASL significantly impaired the antiviral response of goIFNs against TMUV infection *in vitro* (Figures 10C–E). Taken together, our findings suggested that the induction of goMx and goOASL by the goIFN-dependent signaling pathway conferred antiviral and immunomodulatory activities against TMUV infection.

## Discussion

As the first line of defense against invading viruses, interferon is the key component of the innate immune pathway. Generally, IFN-induced signaling cascades are fast and further elicit a positive regulatory feedback loop to enhance the production of IFN or ISGs and boost the antiviral state (35). Moreover, viral infection could effectively trigger the activation of the innate immune response against viral infection. Indeed, a rational hypothesis is that the most highly induced ISGs during infection are the ones that control viral replication (3). In contrast, viruses have the ability to utilize diverse strategies to circumvent the IFN response (37). In this study, the TMUV infection model was first chosen to explore the IFNs involved in the innate immune response *in vivo* and *in vitro*, demonstrating that the mRNA expression level of goIFNs was remarkably induced in immune-related tissue (LI, SP, and T) during TMUV infection (1–4 dpi), and the high expression patterns of the CD8α molecule consisted of the viral location. It is noteworthy that CD8+ T cells mainly produce cytokines, such as IFN-γ and TNF-α, which further activate macrophages and inhibit viral replication (38, 39). We speculated that the TMUV-induced IFN response plays a major role in shaping the adaptive immune response (40, 41). A similar IFN response to TMUV infection was observed in the *in vitro* study, these results suggest that TMUV induced a high expression level of goIFN and activated the IFNβ and IRSE in TMUV-infected GEFs, which was consistent with the previous study (23, 25).

As reported previously, the overexpression of duMAVS increased the expression of IFN and downstream factors and reduced the replication of TMUV (27). However, a study reported that treatment with chicken IFN-α is unable to prevent TMUV infection in DF-1 cells, but mammalian type I IFN can significantly inhibit the replication of TMUV in BHK-21 cells (21). Interestingly, our data indicated that goIFN pre-treatment significantly decreased viral copies and titers, which suggested that goIFNs can inhibit TMUV replication in GEF. This is the first report of avian IFNs that show an inhibition ability in TMUV replication in avian cells. Our previous study revealed that treating GEFs with goIFNs upregulated subsequent ISG expression, such as goIFNs, goMx, and goOASL (36), suggesting that those ISGs may be the workhorses in controlling TMUV infection *in vitro*. With those facts in mind, here, we further investigated the molecular antiviral mechanism of goose type I, II, and III interferon against TMUV.

PBMCs are important immune cells and effectively respond to IFN treatment. Therefore, PBMCs were chosen as cell models and were treated with goIFN at 3 h for detecting the transcriptional profile of immune factors using RNA-seq technology. Surprisingly, the numbers of upregulated and downregulated genes by goIFNλ stimulation were much lower than those regulated by goIFNα or goIFNγ stimulation (Figure 5A). Collectively, the stimulation of goIFNs initiated a series of signaling cascades at early stages, leading to the transcriptional regulation of hundreds of DEGs, and each IFN induced a unique and partially overlapping set of ISGs (Figures 5B,C). Most importantly, we identified approximately 101 IRGs, which were mainly involved in the toll-like receptor signaling pathway, JAK-STAT signaling pathway and antiviral pathway (Figure 6). Moreover, these results were verified by RT-qPCR, and the correlation analysis of RNA-Seq data and qRT-PCR results showed that some relevant relationships between candidate genes may be related to the balance of the host immune response, which remains to be further studied in the future. Previously, human bone marrow macrophages were treated with IFNα and IFNγ for 30 min and functionally validated the antiviral activity of 288 type I and type II ISGs against an RNA virus [vesicular stomatitis virus (VSV)] and a DNA virus (murine gammaherpes virus, MHV-68). Ultimately, 34 ISGs, including OAS1, IFITM3, TRIM25, MDA5, and RIG-I, were identified, which elicited an antiviral effect on the replication of either one or both viruses (42). Moreover, 36 ISGs were tested against West Nile virus (WNV) and dengue virus (DENV) infection, and only five ISGs (IFITM2, IFITM3, ISG20, Viprerin, and PKR) efficiently suppressed WNV and/or DENV (43). Based on those and our data, we assume that goIFNs bind to their cognate receptors in goPBMCs, which trigger the Jak/STAT pathway to stimulate the formation of an IFN-stimulated gene factor 3 (ISGF3) trimer or IFN-γ activation factor (GAF). Subsequently, ISGF3 or GAF translocates into the nucleus and binds to IFN-stimulated response elements or gamma-activated sequence (GAS) promoter elements, resulting in the induction of ISGs, including IFNs, positive regulators (RIG-I, cGAS, PKR), negative regulators (SOCS-1, USP18) and antiviral effectors (Mx, OASL, Viperin, IFITM5, TRIM25) (sketch in Figure 7). The positive regulators could further enhance the PRR signaling pathway, which results in the activation of STING/MAVS and leads to the phosphorylation of interferon response factors 3 or 7 (IRF3/7) or the phosphorylation and ubiquitin-mediated degradation of IκB. Phosphorylated dimers of IRF3/7 or NF-κB translocate into the nucleus, where they bind to and activate specific promoters, triggering the subsequent production of IFNs and ISGs as a positive self-regulatory feedback loop. Consequently, the positive self-regulatory feedback loop greatly enhances the innate immune response against viral infection. Here, we hypothesized that goIFN establishes the cellular antiviral state against TMUV through the induction of ISGs. Based on our data, two important ISGs, goMx, and goOASL, were highly upregulated by three types of goIFNs *in vitro*. With those in mind, goMx and goOASL were considered effective candidates for further antiviral research.

The Mx and OAS families are well-studied proteins in the control of viral infection. Compared with OAS, OASL has additional 2 UBL repeats, which are involved in the initiation of RIG-I-mediated antiviral signaling as a mediator (44). However, only OASL, not OAS, was identified in goose and chicken (45). Our previous research indicated that the overexpression of goOASL significantly reduced the replication of Newcastle disease virus in GEF, and the mRNA expression level of goOASL was significantly increased by TMUV infection *in vivo* (45). Additionally, the antiviral activity of OAS against viruses in the family flavivirus was reported, including hepatitis C virus (HCV) and WNV (46). Mx family members have various antiviral profiles against a wild range of viruses at unique steps in their life cycle (47). However, the number of antiviral studies of bird Mx is still limited and contradictory. In 1995, chicken Mx (chMx) was first identified with no antiviral activity, but chMx was subsequently reported as an antiviral effector against the influenza virus and VSV (48). Moreover, no antiviral effect of duck Mx has been reported (49). In our study, the over expression of goMx and goOASL obviously reduced the replication of TMUV, and subsequent RNAi assay revealed that the knockdown of endogenous goMx and goOASL decreased the antiviral activity of goIFN in controlling TMUV infection. Therefore, we noted that goMx and goOASL were indispensable in goIFN-mediated innate immune responses that restrict TMUV infection. As one of the first characterized antiviral effector pathways, the antiviral activity of OAS has been widely demonstrated *in vitro* and *in vivo* against various viruses (DENV, WNV, HCV, and Japanese Encephalitis virus) through RNase L-dependent and RIG-dependent (RNase L-independent) signaling pathways (44, 50, 51). Reported studies have provided evidence that the Mx GTPase system displayed diverse antiviral activity against many types of viruses at unique steps in their life cycle. Mouse Mx1 inhibited the replication of the influenza virus by blocking viral mRNA synthesis in the nucleus, while human MxA in the cytoplasm prevented the entry of influenza A virus nucleocapsids into the nucleus. However, the molecular mechanism of goMx and goOASL against TMUV infection was uncovered. A further understanding of these ISGs may provide future directions for antiviral therapies against TMUV.

In conclusion, here, goIFN expression was obviously driven by TMUV infection *in vivo* and *in vitro*. Additionally, the antiviral effects of goIFNα, goIFNγ, and goIFNλ against the emerging goose flavivirus TMUV *in vitro* was confirmed for the first time. Based on transcriptome analysis, the gene profile of goIFN-stimulated PBMCs were shown. Subsequently, two important interferon-stimulated genes (ISGs), goMx and goOASL, were identified as the workhorse IFNs in the inhibition of TMUV replication *in vitro* by transient overexpression and knockdown assay. Collectively, these data have prompted the suggestion that TMUV infection-induced goIFN enhanced the antiviral state through their downstream ISGs, which further form a positive regulatory feedback loop to boost the host antiviral effect.

## Ethics Statement

The animal studies were approved by the Institutional Animal Care and Use Committee of Sichuan Agricultural University (No. XF2014-18) and followed the National Institutes of Health guidelines for the performance of animal experiments.

## Author Contributions

SC and WZ designed the experiment. WZ performed the experimental work. WZ and SC wrote the paper. ZW, JZ, MW, RJ, DZ, ML, KS, QY, YW, XC, and AC contributed to analysis the experimental data.

## Conflict of Interest Statement

The authors declare that the research was conducted in the absence of any commercial or financial relationships that could be construed as a potential conflict of interest.

## Supplementary Material

Figure S1 Cytotoxicity of goIFNα, goIFNγ and goIFNλ in goose embryo fibroblasts (GEFs). GEFs were treated with 10 µL of the cell lysates from pcDNA3.1 (+)-goIFNα, goIFNγ, and goIFNλ-transfected BHK-21 cells for 24 h. Similarly, the control group was treated with 10 µL of the cell lysates from pcDNA3.1 (+)-transfected BHK-21 cells. CCK-8 reagent (10 µL) was added into each well for 2 h at 37°C. After that, the plates were evaluated at the 450 nm wavelength with a multi-detection microplate reader. The results were expressed relative to control cells, which were defined as 100% viable.

Figure S2 Pairwise comparison of goIFNα, goIFNγ, and goIFNλ induced IRGs. **(A)** goIFNα vs. goIFNγ; **(B)** goIFNγ vs. goIFNλ; **(C)** goIFNα vs. goIFNλ. Axes represented fold change in response to goIFNα, goIFNγ, or goIFNλ over untreated cells. Red plots indicated goIFNα specific genes. Green plots indicated goIFNγ specific genes. Purple plots indicated goIFNλ specific genes.
