# Supplementary material for: Goose Mx and OASL Play Vital Roles in the Antiviral Effects of Type I, II, and III Interferon against Newly Emerging Avian Flavivirus
Source: Front Immunol. 2017 Aug 23;8:1006. doi: 10.3389/fimmu.2017.01006 (PMC5572330; doi:10.3389/fimmu.2017.01006)
Supplement: Supplementary file 1 [file Table_1.DOCX]

**Table S1 The list of immune-related genes (IRGs).**

| **Gene ID** | **Gene name** | **log2FoldChange**  **(IFNα vs Mock)** | **log2FoldChange**  **(IFNγ vs Mock)** | **log2FoldChange**  **(IFNλ vs Mock)** |
| --- | --- | --- | --- | --- |
| XM_013201722.1 | Actin | -0.18 | 0.06 | 0.09 |
| XM_013200474.1 | ADAR | 1.61 | 1.43 | 0.19 |
| XM_013201875.1 | AKT3 | -0.60 | -0.20 | -0.01 |
| XM_013182642.1 | APAF1 | 0.34 | 0.32 | -0.10 |
| XM_013172978.1 | BID | 3.42 | 3.46 | 1.56 |
| XM_013178135.1 | BIRC2 | 0.72 | 0.51 | -0.18 |
| XM_013188813.1 | C5 | -0.02 | 0.52 | -0.04 |
| XM_013202221.1 | CAPN1 | 0.11 | 0.42 | 0.16 |
| XM_013187585.1 | CASP10 | 0.58 | 1.17 | -0.07 |
| XM_013187588.1 | CASP10 | 0.17 | 1.11 | 0.06 |
| XM_013179825.1 | CASP3 | -0.07 | 0.24 | -0.11 |
| XM_013174578.1 | CASP7 | 1.07 | 0.93 | -0.09 |
| XM_013187192.1 | CBL | -0.26 | -0.08 | 0.06 |
| XM_013200610.1 | CCL19 | 8.19 | 7.04 | 3.21 |
| XM_013181430.1 | CCL20 | 0.02 | 0.54 | -0.33 |
| XM_013189266.1 | CCL4 | 0.04 | -0.23 | -0.42 |
| XM_013189339.1 | CCL4L | 0.53 | 0.66 | -0.33 |
| XM_013189265.1 | CCL5 | 0.32 | 0.11 | -0.19 |
| XM_013198462.1 | CD40 | 0.69 | 0.66 | 0.09 |
| XM_013182918.1 | CD47 | -0.34 | -0.21 | -0.23 |
| XM_013183390.1 | CD58 | 0.32 | 0.22 | -0.17 |
| XM_013194699.1 | CD59 | -0.47 | -0.30 | -0.15 |
| XM_013175979.1 | CD74 | 0.34 | 0.69 | 0.27 |
| XM_013191027.1 | CD93 | -0.40 | -0.40 | -0.19 |
| XM_013187583.1 | CFLAR | 0.37 | 0.43 | 0.01 |
| XM_013172209.1 | cGAS | 1.17 | 0.69 | -0.11 |
| XM_013189456.1 | CIITA | 0.89 | 2.04 | 0.05 |
| XM_013191945.1 | CISH | -0.29 | -0.16 | -0.43 |
| XM_013184365.1 | CSF3 | -0.57 | -1.16 | 0.07 |
| XM_013186335.1 | CXCR4 | -0.06 | -0.07 | -0.18 |
| XM_013199749.1 | DHX58 | 3.04 | 2.21 | 0.19 |
| XM_013194898.1 | FADD | 0.80 | 0.32 | -0.21 |
| XM_013171650.1 | FAS | 0.12 | 0.37 | -0.10 |
| XM_013185550.1 | FOS | 0.28 | 0.03 | 0.09 |
| XM_013198244.1 | HSP90AA1 | 0.43 | 0.10 | -0.05 |
| XM_013183105.1 | HSPA2 | 0.08 | 0.42 | -0.08 |
| XM_013187256.1 | HSPA8 | 0.32 | -0.03 | -0.11 |
| XM_013198319.1 | IFITM5 | 3.16 | 2.31 | -0.06 |
| XM_013175519.1 | IFNGR1 | -0.10 | -0.33 | -0.16 |
| XM_013194016.1 | IFNα | 5.90 | 4.43 | 0.95 |
| XM_013198313.1 | IFNγ | 1.70 | 5.87 | 0.58 |
| XM_013178817.1 | IFNλ | -0.13 | 1.79 | 6.95 |
| XM_013187437.1 | IGSF3 | -0.38 | -0.10 | -0.01 |
| XM_013189617.1 | IKBKE | 0.27 | 0.81 | 0.28 |
| XM_013185532.1 | IkBα | -0.17 | -0.23 | -0.11 |
| XM_013199169.1 | IL1 | -0.56 | -0.64 | -0.38 |
| XM_013178422.1 | IL12 | -0.95 | -0.50 | -0.85 |
| XR_001213371.1 | IL15 | 1.01 | 2.21 | 0.94 |
| XM_013190243.1 | IL1R1 | -0.38 | -0.49 | -0.28 |
| XM_013196285.1 | IL22 | -0.56 | 0.16 | 0.06 |
| XM_013175334.1 | IL22RA2 | -0.04 | -1.44 | -0.03 |
| XM_013180983.1 | IL3R | 0.06 | 0.32 | -0.18 |
| XM_013196876.1 | IL4 | -0.30 | -0.21 | -0.28 |
| XM_013171777.1 | IL6 | 0.72 | 0.06 | -0.28 |
| XM_013181777.1 | IL7R | 0.16 | 0.24 | -0.07 |
| XM_013190619.1 | IL8 | -0.32 | -0.54 | -0.32 |
| XM_013182354.1 | IRAK2 | 0.18 | -0.15 | -0.21 |
| XM_013179816.1 | IRF2 | 1.69 | 1.93 | 0.72 |
| XM_013174398.1 | IRF7 | 2.79 | 3.17 | 0.13 |
| XM_013180585.1 | ITGA2 | -0.34 | -0.45 | -0.19 |
| XM_013171637.1 | ITGA4 | -0.17 | -0.11 | -0.05 |
| XM_013197387.1 | JAK2 | 0.59 | 0.58 | -0.13 |
| XM_013176141.1 | JUN | 0.20 | 0.24 | -0.04 |
| XM_013181636.1 | LIFR | 0.00 | 0.79 | -0.13 |
| XM_013174906.1 | MAP2K3 | -0.61 | -0.23 | -0.30 |
| XM_013191750.1 | MAP3K8 | 0.85 | 0.73 | -0.15 |
| XM_013171142.1 | MDA5 | 3.25 | 3.03 | -0.08 |
| XM_013177828.1 | MED8 | -0.35 | -0.41 | -0.25 |
| XM_013202277.1 | MHC-I | 0.27 | 0.66 | 0.19 |
| XM_013202352.1 | MHC-II | 0.03 | 0.49 | 0.20 |
| XM_013181385.1 | MX | 3.25 | 3.09 | -1.48 |
| XM_013195752.1 | NF-kB | 0.03 | 0.19 | -0.16 |
| XM_013191918.1 | OASL | 4.64 | 4.16 | -0.12 |
| XM_013188118.1 | PER2 | -0.31 | -0.19 | -0.10 |
| Novel00272 | PIM1 | 1.46 | 0.51 | -0.22 |
| XM_013170718.1 | PKR | 3.88 | 3.74 | 0.54 |
| XM_013194076.1 | PML | 3.27 | 2.75 | 0.07 |
| XM_013200056.1 | POLR2 | -0.44 | -0.22 | -0.15 |
| XM_013179156.1 | PRKAR1 | -0.18 | -0.13 | -0.14 |
| XM_013187943.1 | RIG-I | 2.83 | 2.49 | -0.06 |
| XM_013193572.1 | RIPK1 | 1.05 | 0.84 | -0.18 |
| XM_013192412.1 | RIPK2 | 1.60 | 1.30 | -0.15 |
| XM_013189527.1 | SOCS1 | 1.34 | 2.50 | -0.23 |
| XM_013197885.1 | SOCS3 | 0.28 | 0.17 | -0.06 |
| XM_013202364.1 | SOS2 | 0.06 | 0.27 | -0.01 |
| XM_013195524.1 | SRSF4 | -0.40 | -0.14 | 0.13 |
| XM_013187488.1 | STAT1 | 2.29 | 2.28 | 0.06 |
| XR_001214357.1 | STAT2 | 1.97 | 2.32 | 0.05 |
| XM_013201145.1 | STAT6 | -0.32 | 0.04 | 0.11 |
| XM_013170799.1 | TAP2 | 0.85 | 1.09 | 0.16 |
| XM_013185965.1 | TLR1 | 0.72 | 0.49 | -0.21 |
| XM_013175170.1 | TLR2 | 0.61 | 0.14 | 0.00 |
| XM_013181387.1 | TMPRSS2 | 4.04 | 3.07 | 0.12 |
| XM_013195079.1 | TNFSF13 | 0.39 | 0.43 | -0.08 |
| Novel00344 | TPO | 0.21 | -1.01 | -0.19 |
| XM_013178948.1 | TRIM25 | 3.03 | 2.94 | -0.26 |
| XM_013179209.1 | TRIM47 | 1.38 | 0.89 | -0.04 |
| XM_013179202.1 | TRIM65 | 0.83 | 0.77 | 0.17 |
| XM_013172965.1 | USP18 | 4.86 | 3.35 | -0.05 |
| XM_013197215.1 | USP25 | 1.82 | 0.95 | -0.01 |
| XM_013172803.1 | Viperin | 4.77 | 4.23 | -0.41 |
