# Supplementary material for: Goose Mx and OASL Play Vital Roles in the Antiviral Effects of Type I, II, and III Interferon against Newly Emerging Avian Flavivirus
Source: Front Immunol. 2017 Aug 23;8:1006. doi: 10.3389/fimmu.2017.01006 (PMC5572330; doi:10.3389/fimmu.2017.01006)
Supplement: Supplementary file 2 [file Table_2.DOCX]

**Table S2 Gene regulation of top up-regulated genes (p-adj < 0.05) between IFNα and mock group.**

**Gene ID Gene Name Log2 Fold Change P-value Q-value (p-adj)**

XM_013200610.1 CCL-19 8.1932 3.12E-50 1.36E-47

XM_013181201.1 IFT27-2B 6.1719 1.64E-73 1.24E-70

XM_013194152.1 IFIT-5 6.1186 1.71E-13 2.84E-13

XM_013194016.1 IFNα 5.8978 1.82E-14 1.84E-12

XM_013172592.1 CMPK2 4.9468 3.30E-16 8.75E-16

XM_013172965.1 USP18 4.8562 1.25E-11 1.66E-11

XM_013172803.1 Viperin 4.7740 1.89E-15 3.85E-14

XM_013191918.1 OASL 4.6389 5.37E-77 4.19E-74

XM_013178947.1 TRIM25 4.6254 6.42E-40 1.85E-37

XM_013193367.1 KCNK9 4.5473 0.00152 0.0351
