# Supplementary material for: Goose Mx and OASL Play Vital Roles in the Antiviral Effects of Type I, II, and III Interferon against Newly Emerging Avian Flavivirus
Source: Front Immunol. 2017 Aug 23;8:1006. doi: 10.3389/fimmu.2017.01006 (PMC5572330; doi:10.3389/fimmu.2017.01006)
Supplement: Supplementary file 3 [file Table_3.DOCX]

**Tabel S3 Gene regulation of top up-regulated genes (p-adj < 0.05) between IFNγ and mock group.**

**Gene ID Gene Name Log2 Fold Change P-value Q-value (p-adj)**

XM_013200610.1 CCL-19 7.0391 1.13E-21 2.28E-19

XM_013194152.1 IFIT-5 5.0991 1.95E-45 1.28E-42

XM_013194016.1 IIFNα 4.4328 2.90E-12 3.07E-10

XM_013178947.1 TRIM25 4.3949 2.69E-31 8.33E-29

XM_013172592.1 CMPK2 4.3076 6.27E-64 9.93E-61

XM_013181201.1 IIFT27-2B 4.2557 2.82E-14 3.47E-12

XM_013172803.1 Viperin 4.2323 5.26E-47 3.54E-44

XM_013191918.1 OASL 4.158 9.54E-48 6.76E-45

XM_013172965.1 USP18 3.3527 4.27E-31 1.29E-28

XM_013170960.1 Mx 3.2551 1.26E-83 2.83E-80
