# Supplementary material for: Goose Mx and OASL Play Vital Roles in the Antiviral Effects of Type I, II, and III Interferon against Newly Emerging Avian Flavivirus
Source: Front Immunol. 2017 Aug 23;8:1006. doi: 10.3389/fimmu.2017.01006 (PMC5572330; doi:10.3389/fimmu.2017.01006)
Supplement: Supplementary file 4 [file Table_4.DOCX]

**Tabel S4 Gene regulation of top up-regulated genes (p-adj < 0.05) between IFNλ and mock group.**

**Gene ID Gene Name Log2 Fold Change P-value Q-value (p-adj)**

XM_013178817.1 IFNλ 6.9501 2.99E-23 8.25E-19

XM_013191374.1 PAPPA2 5.2835 9.03E-06 0.002075

XM_013201514.1 TMEM79 5.1898 1.92E-05 0.004081

XM_013171470.1 ZP4 4.4920 0.00041876 0.045558

XM_013170917.1 MYO7A 3.3453 6.52E-06 0.001676

XM_013188704.1 ADGRD2 3.1435 8.55E-05 0.013308

XM_013193904.1 Hmcn2 3.0199 0.00020459 0.027113

XM_013188227.1 AMER2 2.9019 6.67E-05 0.010941

XM_013190739.1 CELSR1 2.6698 6.66E-05 0.010941

XM_013183078.1 KIF26 3.2551 0.00018975 0.025391
