# Supplementary figures and images for: Goose Mx and OASL Play Vital Roles in the Antiviral Effects of Type I, II, and III Interferon against Newly Emerging Avian Flavivirus
Source: Front Immunol. 2017 Aug 23;8:1006. doi: 10.3389/fimmu.2017.01006 (PMC5572330; doi:10.3389/fimmu.2017.01006)

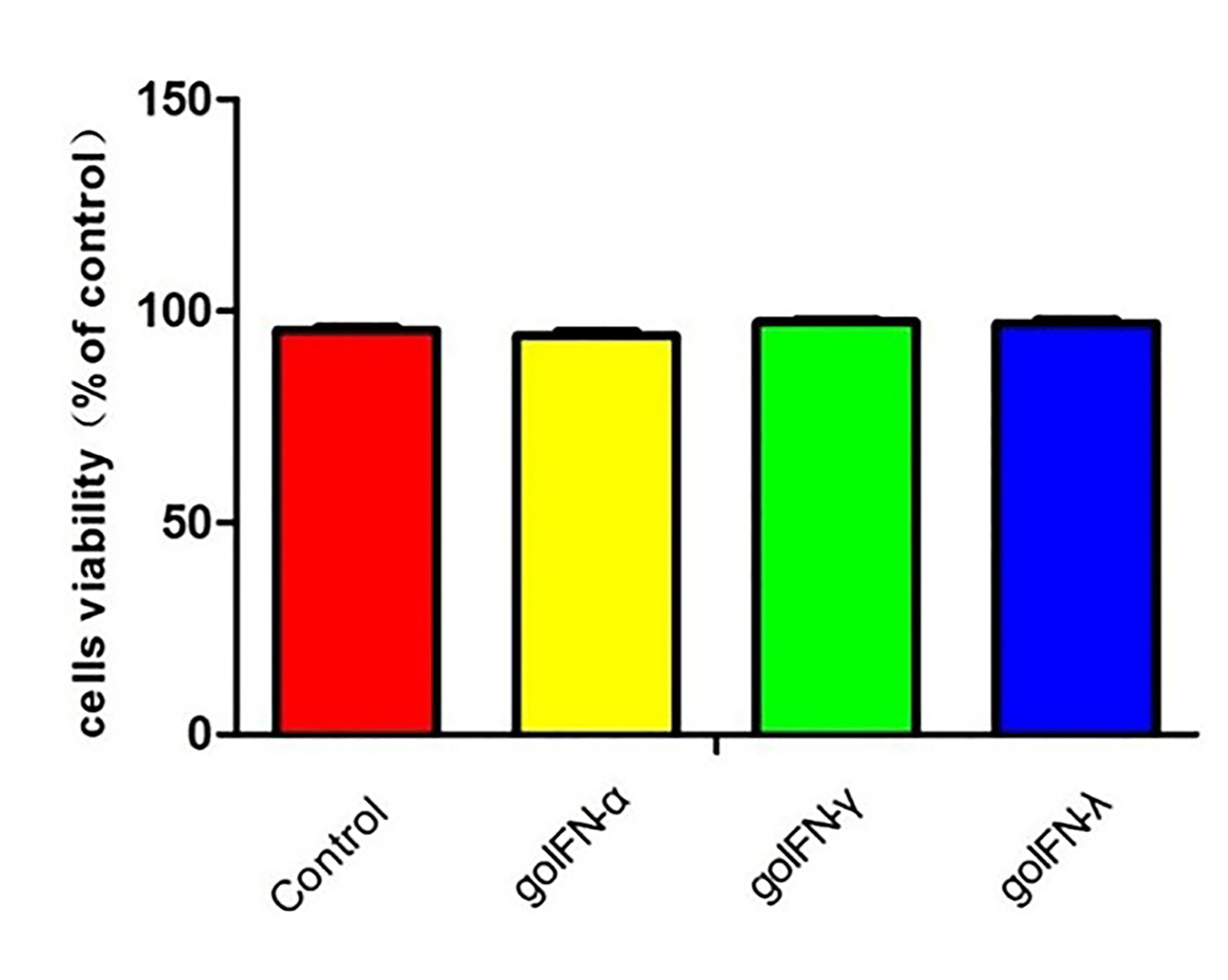

Supplement: Figure S1 — Cytotoxicity of goIFNα, goIFNγ and goIFNλ in goose embryo fibroblasts (GEFs). GEFs were treated with 10 µL of the cell lysates from pcDNA3.1 (+)-goIFNα, goIFNγ, and goIFNλ-transfected BHK-21 cells for 24 h. Similarly, the control group was treated with 10 µL of the cell lysates from pcDNA3.1 (+)-transfected BHK-21 cells. CCK-8 reagent (10 µL) was added into each well for 2 h at 37°C. After that, the plates were evaluated at the 450 nm wavelength with a multi-detection microplate reader. The results were expressed relative to control cells, which were defined as 100% viable. [file Image_1.JPEG]

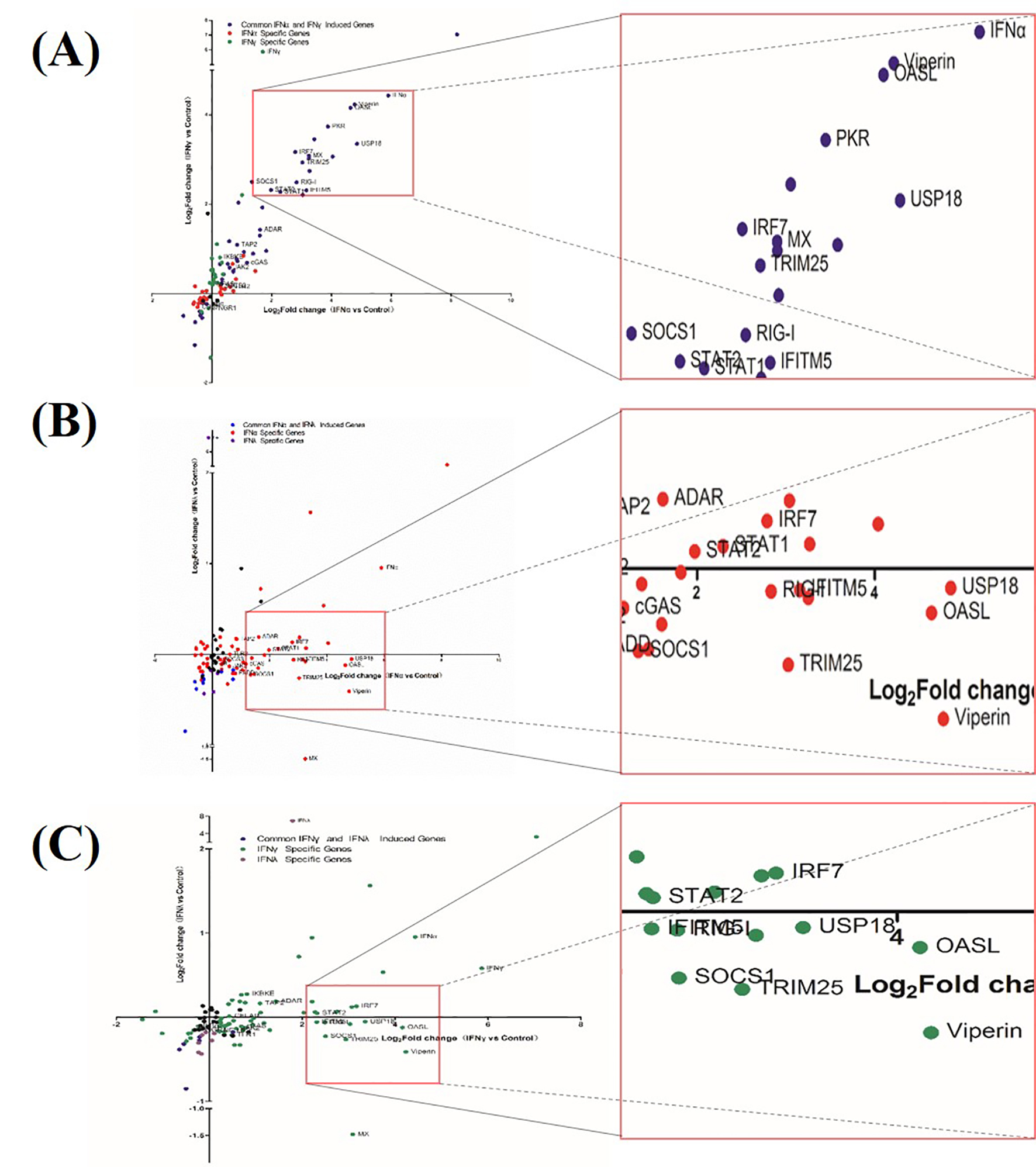

Supplement: Figure S2 — Pairwise comparison of goIFNα, goIFNγ, and goIFNλ induced IRGs. (A) goIFNα vs. goIFNγ; (B) goIFNγ vs. goIFNλ; (C) goIFNα vs. goIFNλ. Axes represented fold change in response to goIFNα, goIFNγ, or goIFNλ over untreated cells. Red plots indicated goIFNα specific genes. Green plots indicated goIFNγ specific genes. Purple plots indicated goIFNλ specific genes. [file Image_2.JPEG]
